# Outpatient management of chronic expanding subdural hematomas with endovascular embolization to minimize inpatient admissions during the COVID-19 viral pandemic

**DOI:** 10.1177/1591019921996510

**Published:** 2021-02-16

**Authors:** Pouya Entezami, Nicholas C Field, John C Dalfino

**Affiliations:** Department of Neurosurgery, 138207 Albany Medical Center, Albany, NY, USA

**Keywords:** Chronic subdural hematoma, COVID-19, middle meningeal artery embolization, outpatient

## Abstract

Chronic subdural hematomas are complex collections that usually form after a trauma, particularly in elderly patients. This vulnerable population is at increased risk given the current viral pandemic. We share our experience in managing minimally symptomatic, enlarging subdural collections via middle meningeal embolization through the outpatient setting. This approach minimizes inpatient hospitalizations in hopes or reducing nosocomial spread (e.g., of COVID-19).

## Introduction

Chronic subdural hematomas (SDH) are typically found on follow-up imaging of known acute SDHs or through evaluations for headaches or falls, particularly in elderly patients.^1–3^ Expanding collections often require inpatient management in symptomatic patients. However, with the current SARS-CoV-19 (COVID-19) pandemic, our practice has increasingly shifted the management of mildly symptomatic patients with minimal hematoma expansion to the outpatient setting to avoid inpatient hospitalization.

Patients with large symptomatic lesions may be offered a procedure for hematoma evacuation, either through bedside evacuation using a small burr hole, or an operation via burr hole(s) or traditional craniotomy.^4,5^ Conversely, patients with small, minimally symptomatic lesions can be observed with serial imaging in the hope that the collections will spontaneously resorb.

The most difficult treatment decisions we commonly encounter are:

1. Large or growing collections that are minimally symptomatic.
2. Small collections associated with persistent, severe headaches.
3. Patients with subdural collections who require antithrombotic therapy.

In our practice (and in other centers around the country) we have increasingly used embolization of the middle meningeal artery (MMA) as the sole treatment for chronic expanding SDHs.^6–13^ The premise is based on the observation that the outer membrane of a chronic SDH parasitizes its blood supply from the MMA. Subsequently, embolization of this artery reduces the rate of flow through this fragile and porous vascular network and prevents further microhemorrhage and transudation of blood and fluid into the subdural space.^1,14^ The collection is then reabsorbed slowly over time, in many cases without surgical drainage. We present five patients in which we have managed a chronic SDH by embolization of the MMA as the sole treatment through the outpatient setting (Table 1), as proof of concept that this can be done safely. Avoiding an inpatient admission allows us to minimize the risk to the patient of an admission in the setting of COVID-19. All embolization procedures were performed under moderate conscious sedation via a transfemoral approach.

**Table 1.** Patient summary.

Case	Presenting symptom	Anti-thrombotic/Anti-coagulant	Pre-embolization treatments	Indication
#1	Imaging follow-up	Apixaban	None	↑ Size, HA
#2	↑ HA	None	SEPS	↑ Size
#3	Falls, HA	Aspirin, Clopidogrel	None	↑ Size, fatigue
#4	Falls, HA	None	None	Severe HA
#5	Seizure	None	None	↑ Size, HA
#6	HA	Clopidogrel	None	Recurrent bleed

HA: headache; SEPS: subdural evacuating port system.

## Case presentations

**Figure 1.**
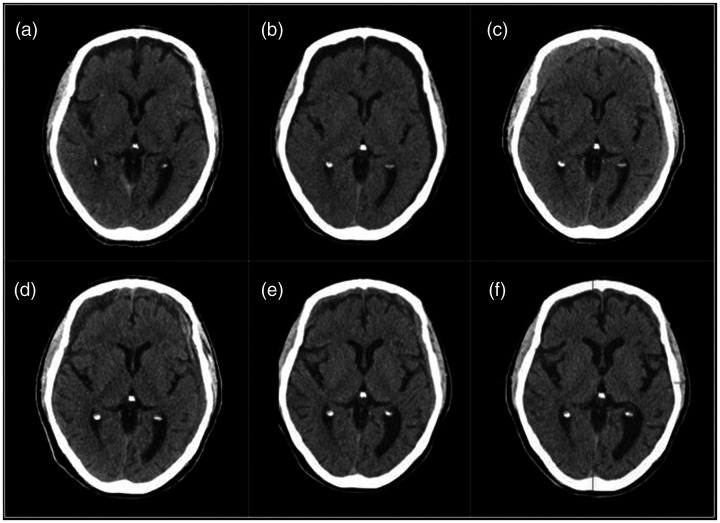
Forty-seven year old (a) initially with a small SDH on CT, (b) showing slight enlargement after three weeks. (c) Acute blood developed once anticoagulation was restarted. MMA embolization was performed and imaging at (d) 6 weeks, (e) 5 months, and (f) 12 months post-embolization showed resolution.

Case 1: A 47 year old with history of seizure disorder and alcohol abuse, on anticoagulation (apixaban), presented to the Emergency Department after a fall. He had no focal clinical deficits, but computed tomography (CT) for trauma workup revealed a small acute SDH (Figure 1(a)). Anticoagulation was held and the patient was discharged after observation. He was rescanned 3 weeks later showing enlargement of the collection (Figure 1(b)). A third scan 1 week later was stable (not shown) and the patient was restarted on apixaban. However, the patient returned one month later complaining of headaches, with acute blood on imaging (Figure 1(c)). Anticoagulation was again stopped and the patient was offered burr hole drainage, but refused. He was referred for MMA embolization. The embolization was performed under conscious sedation as an outpatient admission. He patient was discharged 2 hours post-procedure after resuming anticoagulation. Follow up imaging at 6 weeks, 5 months, and 12 months post-embolization showed resolution (Figure 1(d) to (f)). No further treatment was required.

**Figure 2.**
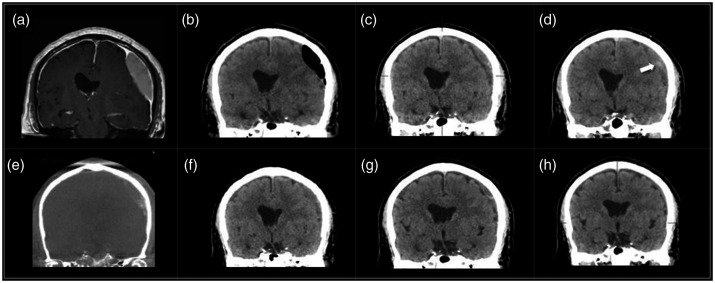
Sixty-one year old (a) with a chronic SDH over the left convexity on MRI, (b) drained via a twist drill craniotomy. (c) Recurrence was seen after one month, (d) with enlargement after two weeks. Embolization was performed, (e) with contrast staining of the collection following the procedure. Progressive reabsorption at (f) 1, (g) 3, and (h) 6 months is shown.

Case 2: A 61 year old male was evaluated by a neurologist after complaining of headaches for 3 weeks. Magnetic resonance imaging (MRI) confirmed a chronic SDH over the left convexity (Figure 2(a)). The collection was drained via a twist drill craniotomy (Figure 2(b)). One month after drainage the patient continued to have headaches and a recurrent SDH was found (Figure 2(c)). Follow up CT two weeks later showed slight enlargement of the collection and increased midline shift (Figure 2(d)). Embolization of the left MMA was performed, with contrast-staining of the collection following the procedure (Figure 2(e)). Follow up imaging at 1, 3, and 6 months (Figure 2(f) to (h)) show progressive reabsorption.

**Figure 3.**
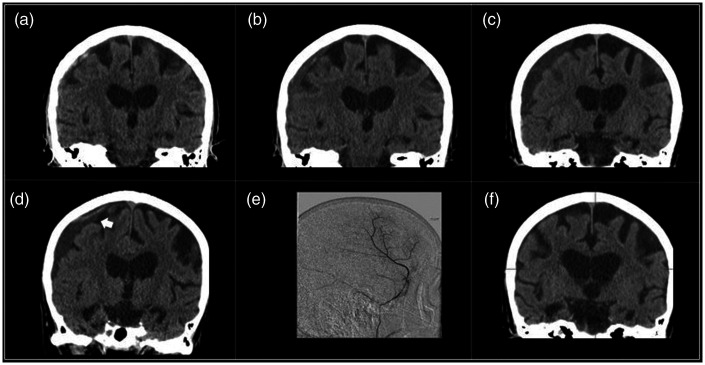
Eighty-seven year old (a) with mixed density SDH, enlarging at (b) 1 and (c) 3 weeks, and (d) ultimately developing a new punctate hemorrhage. (e) Embolization of the MMA was performed, (f) with resolution of the collection at one month.

Case 3: An 87 year old on aspirin and clopidogrel for coronary artery disease complained of headaches, recurrent falls, and cognitive decline, leading to discovery of a small mixed density SDH (Figure 3(a)). Antiplatelet agents were held, but progressive enlargement was seen at 1 and 3 weeks (Figure 3(b) and (c)). At 4 weeks the patient developed vomiting and fatigue and a new punctate hemorrhage was found (Figure 3(d)). Embolization of the MMA was performed (Figure 3(e)). Imaging 1 month post-procedure showed complete resolution of the subdural collection (3F). Antiplatelet agents were resumed.

**Figure 4.**
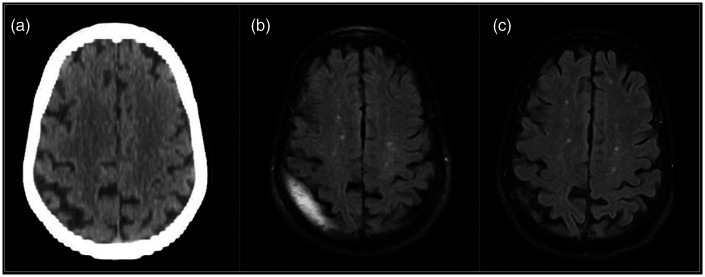
Seventy-eight year old (a) with a negative outside hospital CT following trauma but (b) a small subdural collection on MRI. (c) Collection resolved on imaging two months following MMA embolization.

Case 4: A 78 year old female fell on her assist scooter while recovering from an ankle injury. She suffered no loss of consciousness or apparent neurological injury but suffered headaches for a week following. While her CT remained negative (Figure 4(a)), a follow-up MRI for continued intractable headaches revealed a small subdural collection (Figure 4(b)). Embolization of the right MMA was performed. The patient had immediate headache relief. The patient’s symptoms and collection (Figure 4(c)) were both resolved at 2 month follow-up.

**Figure 5.**
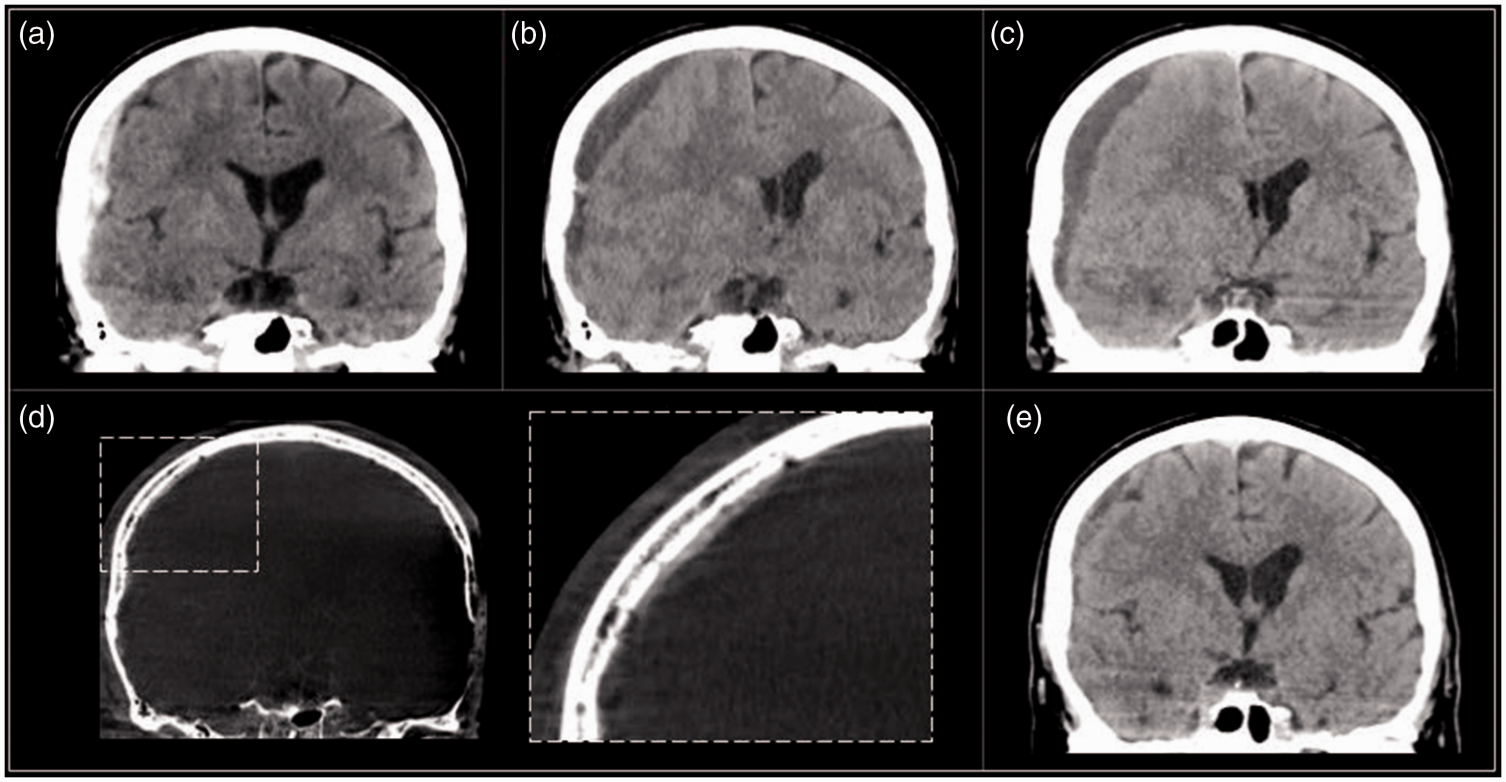
Seventy year old (a) with mixed density right convexity SDH, showing growth and maturation of the collection (b) at two weeks and (c) several months later. Embolization was performed (d to e) with contrast staining seen following the procedure, (f) and post-procedure CT 3 weeks later showed substantial improvement.

Case 5: 70 year old was admitted through the ED with a single, brief episode of aphasia and dysarthria. The patient reported a fall 5 days prior to admission. CT revealed a mostly acute appearing right convexity SDH (Figure 5(a)). He was started on levetiracetam and discharged home 2 days later. He had a follow up scan 2 weeks later showing growth and maturation of the collection (Figure 5(b)). He developed progressive headaches and CT scan several months later revealed enlargement of the collection (Figure 5(c)). Embolization was performed (Figure 5(d) to (e)) and post-procedure CT 3 weeks later showed substantial improvement (Figure 5(f)).

## Discussion

Mixed density hemorrhagic collections over one or both convexities are commonly discovered on outpatient imaging performed for the workup of headaches or other neurological complaints. These chronic SDHs are typically traced back to a fall or other minor trauma.^2^ A patient with a small collection and minor or resolving symptoms may be managed expectantly with serial imaging, as in many cases these collections will resolve, on their own while those with large symptomatic lesions are typically offered surgical drainage.^1^

In some cases, patients with SDHs that were initially small will demonstrate growth of the collection over time rather than resolution, requiring treatment. The source of growth is attributed to microhemorrhages from the fragile vascular network found in the outer membrane.^1,15,16^ The events that lead to the formation of a chronic SDH are still debated (we prefer the model proposed by Haines et al.^17^), but one thing is certain: every expanding chronic SDH is made up of two parts, (1) the initial, evolving hemorrhage and (2) the continuing hemorrhage.^1^

Surgical drainage – via either a burr hole craniotomy or mini-craniotomy – can leave behind most, if not all, of the vascular outer membrane. Predictably, this method carries a high rate of recurrence.^5,11,18^ Embolization, on the other hand, can prevent ongoing hemorrhage of the outer membrane by disconnecting this blood supply, allowing for spontaneous reabsorption of the collection over time of clots that were previously expanding in the outpatient setting. This is particularly helpful in patients requiring antithrombotic therapy, as seen in our cohort.

In our practice, we consider outpatient embolization for patients who present with mild symptoms (such as headaches, gait imbalance, mild weakness, etc.) who are in a safe living environment. The procedure is well tolerated with small doses of versed and fentanyl for sedation. Local sedation is given to the puncture site as well. We use particles for our embolic material, and close with either an Angioseal if done via groin puncture or a TransRadial (TR) band if performed transradially (Terumo Interventional Systems, Somerset, NJ, USA). We monitor the patients for 2 hours post-operatively, examining them for neurological changes (particularly concerning vision) or procedural complications (such as groin/wrist hematoma, progressive headaches, nausea, vomiting, or medication reactions) prior to discharging them home from our post-procedural area. While the outpatient plan is discussed prior to the procedure, should the patient feel uncomfortable about discharge or develop signs of complications afterwards they would be admitted.

Middle meningeal artery embolization is relatively painless and can be done under conscious sedation with minimal risk of delayed complications.^6,7^ This procedure is amenable to a transradial approach in select patients, furthering its potential as an outpatient procedure. In our experience, MMA embolization can be performed safely as an outpatient procedure. Furthermore, eliminating the overnight (or longer) inpatient stay following surgical SDH evacuation improves patient comfort and may reduce the risk of iatrogenic complications, especially in an era where patients are at risk of contracting viridae such as COVID-19 from interactions within the hospital.

COVID-19 has severely limited the resources and hospital capacity available for the managing non-urgent surgical diseases across subspecialties.^19,20^ Elderly patients – the same ones who are at most risk for developing chronic SDHs – are at increased risk from the virus, and efforts should be made to limit their time as an inpatient due to the risk of nosocomial spread.^21–24^ The lessons learned here may encourage providers to employ similar principles to endovascular procedures and consider outpatient treatment for other diagnosis as well (given appropriate monitoring protocols).

## Conclusion

Our initial impression of MMA embolization as a primary treatment for chronic expanding subdural hematomas in the outpatient setting is that it is safe. Given the current viral pandemic, outpatient management of elderly patients is an attractive option, providing neurosurgeons the ability to procedurally manage patients with progressive hemorrhage while minimizing or eliminating the length of inpatient admission.
